# Gatm ablation disrupts spermatogenesis by impairing ribosome biogenesis and coupling defective steroid biosynthesis to immunoglobulin silencing

**DOI:** 10.3389/fcell.2026.1786659

**Published:** 2026-03-12

**Authors:** Shun Li, Lixiang Chen, Mengmin Zhu, Lin Yang, Hanqing Wu, Xiaohui Zhou

**Affiliations:** 1 Department of Animal Model, Shanghai Public Health Clinical Center, Fudan University, Shanghai, China; 2 School of Life Sciences, Shanghai Normal University, Shanghai, China

**Keywords:** cacospermia, creatine metabolism, GATM, immunoglobulin silencing, male infertility, ribosome biogenesis, spermatogenesis, testicular transcriptome

## Abstract

**Background:**

Glycine amidinotransferase (GATM) catalyzes the rate-limiting step in creatine biosynthesis, a pathway essential for cellular energy buffering in tissues with high metabolic demands. While its roles in muscle and brain are well established, the function of GATM in testicular development and spermatogenesis remains largely unexplored.

**Methods:**

We generated a constitutive *Gatm* knockout mouse model using CRISPR/Cas9 technology. Comprehensive phenotypic characterization was performed through histological analysis, transmission electron microscopy (TEM), and transcriptomic profiling of whole testes and purified spermatozoa from *Gatm*, heterozygous (*Gatm*), and wild-type (WT) mice.

**Results:**

*Gatm* deficiency resulted in severe testicular atrophy, disorganized seminiferous tubules with epithelial thinning (reduced to 2–3 layers), and interstitial edema. Spermatogenesis was arrested, leading to dramatically reduced sperm density. Ultrastructural analysis revealed hallmark features of cacospermia, including mitochondrial depletion in the midpiece and nuclear vacuolization. Transcriptomic profiling demonstrated widespread dysregulation: testicular tissues showed downregulation of ribosome biogenesis and mitochondrial complex assembly pathways, whereas mature spermatozoa exhibited impaired steroid biosynthesis and ion transport. Notably, multiple immunoglobulin variable region genes (e.g., *Ighv14-1*, *Igkv19-93*) were completely transcriptionally silenced specifically in *Gatm*-deficient testes.

**Conclusion:**

Our findings establish GATM as a multifunctional regulator of spermatogenesis, integrating creatine-dependent energy metabolism with translational capacity, organelle architecture, and immune-related gene regulation. The coordinated disruption of mitochondrial and ribosomal pathways provides a novel mechanistic framework for understanding the metabolic origins of male infertility.

## Introduction

Creatine phosphate serves as a pivotal energy shuttle, maintaining ATP homeostasis in tissues with high metabolic demands such as skeletal muscle, brain, and testes (Wallimann et al., 2011). The biosynthesis of creatine is initiated by glycine amidinotransferase (GATM), a mitochondrial enzyme that catalyzes the transfer of an amidino group from arginine to glycine, producing guanidinoacetate—the direct precursor of creatine (Yu et al., 2022). While GATM is best known for its essential role in energy metabolism, emerging evidence highlights its involvement in a wide spectrum of physiological and pathological processes. Notably, GATM has been identified as a key mitochondrial hub gene in prostate cancer (Liu et al., 2026), suggesting a potential role in tumorigenesis and cancer cell metabolism. Genome-wide association studies have also linked GATM genetic variants to sarcopenia-related traits (Zhu et al., 2025), implicating its influence on age-related muscle loss. Furthermore, Shriver et al. (Shriver et al., 2025) reported that GATM functions as a host dependency factor for influenza A virus (IAV) infection in both human cells and mouse models; knockdown of Gatm significantly attenuates viral pathogenesis and dampens excessive host inflammatory responses following infection. Beyond these roles, GATM has been implicated in the regulation of pain susceptibility through mitochondrial gene networks (Liu, 2025), highlighting its contribution to neurosensory pathways. Additionally, dysregulation of GATM expression or activity has been associated with chronic kidney disease (CKD) (Liu et al., 2024; Medeiros et al., 2025) and atherosclerosis (AS) (Liu et al., 2025), further underscoring its systemic impact on metabolic and cardiovascular health. Collectively, these findings position GATM not only as a cornerstone of cellular energy metabolism but also as a multifaceted regulator across diverse disease contexts—from infectious diseases and cancer to aging and chronic degenerative conditions. However, its functional roles in reproductive biology remains incompletely understood.

Spermatogenesis involves tightly regulated phases of mitotic proliferation, meiotic division, and post-meiotic differentiation—collectively representing one of the most energy-intensive processes in mammals (Wei et al., 2022; Li and Albertini, 2013). Mitochondria function as central hubs in this energy-demanding process, not only generating ATP but also regulating redox balance, calcium signaling, and apoptosis during germ cell development (Placidi et al., 2022; Schirrmacher, 2020; Kang et al., 2024; Peng et al., 2022). GATM, a mitochondrial gene (Liu, 2025; Liao et al., 2023; Carney, 2018; Forst et al., 2021), warrants further investigation regarding its role in reproduction.

In this study, we established a constitutive *Gatm* knockout mouse model using CRISPR/Cas9-mediated genome editing. Through an integrative approach combining histology, ultrastructural imaging, and multi-level transcriptomic analyses of whole testes and purified spermatozoa, we systematically assessed the impact of *Gatm* ablation on testicular development and sperm function.

Our results reveal that *Gatm* deletion leads to profound testicular atrophy, disorganized seminiferous tubules, and complete spermatogenic arrest, culminating in severely diminished sperm count and motility. Ultrastructural abnormalities characteristic of cacospermia—including mitochondrial loss in the flagellar midpiece and nuclear vacuolization—were observed in mutant sperm. Transcriptome-wide analyses uncovered broad pathway-level disruptions: ribosome biogenesis and mitochondrial complex assembly were significantly downregulated in testicular tissue, while steroid biosynthesis and ion transport were impaired in mature sperm. Importantly, we detected specific and complete transcriptional silencing of multiple immunoglobulin variable region genes (*Ighv*, *Igkv*) in *Gatm*-deficient testes—a phenomenon independent of somatic contamination or global transcriptional shutdown.

Collectively, these findings position GATM as a pleiotropic guardian of male fertility, functioning beyond its canonical metabolic role to coordinate translational output, organelle integrity, and local immune quiescence during germ cell development. These data illustrate how perturbation of a single metabolic node can propagate failure across multiple regulatory tiers. We propose that GATM acts as a metabolic gatekeeper ensuring niche stability in the testis, offering a mechanistic paradigm for how metabolic defects drive complex reproductive disorders through coupled deterioration of metabolic, structural, and immune functions.

## Materials and methods

### Mouse *Gatm* gene and protein sequences analysis

Nucleic acids and amino acids sequences of mouse Gatm were searched and downloaded from NCBI GenBank data-base. Nucleotide BLAST was used for sequences alignment and Open Reading Frame Finder was applied for gene coding region searching. The conserved domains of mouse Gatm protein was analyzed by NCBI Conserved Domains. The transmembrane region of mouse Gatm protein was predicted by using TMHMM server. SWISS-MODEL was used for constructing the 3D crystal structure of mouse Gatm protein.

### Generation and genotyping of *Gatm* gene knockout mice

Gene targeted sites for mouse Gtam were analyzed by the CHOPCHOP (http://chopchop.cbu.uib.no/). The gene targeted sites without predicted off-targeted sites and with highest score were chosen. The methods of generation gene knockout mice by using CRISPR/Cas9 system were described as previous publication (Qin et al., 2016; Wang et al., 2013). Detailed experimental procedures were provided in the Supplementary Material. The total time required to generate and genotype the stable Gatm^−/−^ line was approximately 8–12 months, including embryo microinjection, F0 generation breeding, F1 genotyping, and establishment of homozygous breeding colonies. For breeding, we adopted an optimized strategy to generate experimental animals: GATM^−/−^ females were crossed with GATM^+/−^ males. This cross yields, on average, 50% GATM^−/−^ and 50% GATM^+/−^ offspring. GATM^−/−^ male mice were then selected from the GATM^−/−^ offspring cohort for downstream experiments.

### Ethics statement and sample preparation

Ethical approval was obtained from the Research Ethics Committee of Shanghai Public Health Clinical Center under permission number 2020-A039-01. All mice were housed at the animal facilities in Shanghai Public Health Clinical Center under specific pathogen-free (SPF) conditions in individually ventilated cages (IVCs) with a 12-h light/dark cycle, controlled temperature (22 °C–24 °C), and relative humidity (40%–70%). Animals had *ad libitum* access to standard chow and water. Breeding pairs were maintained at a 1:1 sex ratio (male:female). All experiments were performed under consistent SPF conditions to minimize environmental variability. A total of 68 mice were used in this study, including: 18 wild-type (WT) males, 28 *Gatm* ^−/−^ males, and 22 *Gatm* ^+/−^ males. The samples of testis were collected for further study.

### Histopathological analysis

Mice were humanely euthanized by carbon dioxide asphyxiation. For histological pathology, the testes from Gatm^−/−^, Gatm^+/−^ and litter mate control wild-type mice were collected and fixed in 4% formalin solution overnight and embedded in paraffin. 4-μm-thick sections were sliced and stained with hematoxylin and eosin (H&E).

### Electron microscope

The testes from Gatm^−/−^ and litter mate control wild-type mice were collected and quickly put into 2.5% glutaraldehyde fixed solution at 4 °C for more than 3 h. All the samples were washed by 0.1 mol L^-1^ phosphoric acid buffer solution for three times, followed by shaking and fixing with 0.5% osmic acid at 4 °C for 3 h. Then, samples were washed with buffer solution for three times, and dehydrated step by step with ethanol and followed by epoxypropane. The samples were soaked and embed by using Spurr resin, and the embedded block was placed on an ultrathin microtome (Leica UC7) for sectioning. The ultrathin sections were 70 nm thick and stained by uranyl acetate and lead citrate. All the sections were observed and photographed under a transmission electron microscope (ThermoFisher Talos120).

### Serum hormone measurement

Blood samples were collected from the orbital sinus of 12-month-old Gatm^−/−^ and wild-type (WT) male mice. Whole blood was allowed to coagulate at room temperature for 2 h, then centrifuged at 3,000 *g* for 15 min at 4 °C to separate serum. Serum was carefully aliquoted into sterile tubes and stored at −80 °C until analysis to avoid repeated freeze-thaw cycles. Serum levels of testosterone, follicle-stimulating hormone (FSH), and luteinizing hormone (LH) were quantified using enzyme-linked immunosorbent assay (ELISA) kits purchased from Elabscience Biotechnology Co., Ltd., (Wuhan, China): Mouse FSH ELISA Kit (Cat. No. USCN-CEA830Mu), Mouse LH ELISA Kit (Cat. No. CEA441Mu), Mouse Testosterone ELISA Kit (Cat. No. CEA458Ge). All assays were performed strictly according to the manufacturers’ instructions. Optical density (OD) was measured at 450 nm using a microplate reader (BioTek Epoch, United States). Hormone concentrations were calculated from standard curves generated with serial dilutions of recombinant mouse standards provided in each kit.

### Testes and sperms collection for RNA-sequencing

Mice were humanely euthanized by CO_2_‚ asphyxiation using a gradual fill method (flow rate: 30% of chamber volume per minute) followed by cervical dislocation to confirm death, in accordance with the Laboratory animal-Guidelines for euthanasia GBT39760-2021. The abdominal skin was disinfected with 75% ethanol. The skin and peritoneum were sequentially incised with surgical scissors to expose the bilateral testes. The testes were directly used for RNA extraction and sequencing. In addition, the epididymal cauda, located dorsally to the testes and presenting as an inflated elliptical structure connected to the vas deferens, was identified. The epididymal cauda and vas deferens were lifted using surgical forceps, and surrounding adipose and ligament tissues were removed with surgical scissors. The isolated tissues were placed in a Petri dish containing 200 μL of PBS. Under a stereomicroscope, residual adipose tissue and blood vessels on the surface were meticulously dissected using microforceps. The tissues were rinsed three times with PBS, and the supernatant was discarded. Subsequently, 200 μL of fresh PBS was added to the dish. The epididymal cauda was punctured with a 1 mL syringe needle to release sperm from both the epididymal cauda and vas deferens. The resulting suspension was collected into a 1.5 mL centrifuge tube and centrifuged at 3,000 rpm for 10 min. After discarding the supernatant, the pellet was resuspended in Trizol for subsequent RNA extraction and sequencing.

### RNA isolation, library construction and Illumina sequencing

For each sample, the total RNA was isolated by using the RNA mini kit (Qiagen, Germany). The concentration of RNA was measured by Qubit (Thermo, Waltham, MA, United States), and their quality were examined by agarose gel electrophoresis. The high quality RNA was applied for RNA-seq library construction by using the TruSeq RNA Library Prep Kit V2. The libraries were then pooled and sequenced on NovaSeq 6000 (Illumina) at a depth of approximately 30 M reads per sample, and the 150 bp paired-end reads were generated for further analyses.

### Quality control and reference genome alignment

To ensure the quality of sequencing data can be used for downstream analysis, the software of Skewer (v0.2.2) was used to analyze and remove the sequence reads with adapter and low-quality fragments. FastQC software (v0.11.5) was used for quality control analysis of the preprocessed data and calculated the base ratio of Q20 and Q30. For each sample, the software of Bowtie2 was used to align the preprocessed sequence with the reference genome sequence of the corresponding sequenced species. The reference genome of mouse was Mouse genome mm10.

### Quantification of overall gene expression levels

For all samples, the software of StringTie was used to count the read numbers and map reads to genes. The expression level of each gene was calculated by using FPKM (the fragments per kilobase of exon per million mapped fragments). FPKM is currently the most commonly used quantitative expression index in paired-end transcriptome sequencing, which takes into account the effects of gene length and sequencing data volume. The calculation formula of FPKM is total fragments dividing mapped reads (millions) and exon length (KB). The exon length of a gene was defined as the sum of the lengths of the exon non-redundant regions of known transcripts within the gene. Mapped reads Defined as the total number of sequence pairs aligned to the reference genome sequence. Generally, if the value of FPKM for a gene is greater than 0.1, the gene is thought to be expressed in the sample.

### Differential expression genes (DEGs) analyses

For differential expression genes screening between the groups of Gatm knockout and wild type, the DESeq2 v1.16.1 package in R software was used. DESeq2 v1.16.1 serves statistical algorithms for determining differential gene expression from gene expression data through the model based on a negative binomial distribution. The *p* values were adjusted by Benjamini and Hochberg’s correction for controlling the FDR (false discovery rate). Genes with a corrected p value less than 0.05 and log2FoldChange more than 1 screened by DESeq2 v1.16.1 were defined as DEGs.

### GO and KEGG enrichment analysis for DEGs

For DEGs, Gene ontology (GO) was analysed by using topGO (http://www.bioconductor.org/packages/release/bioc/html/topGO.html) and the website (http://www.genome.jp) was applied for analysing the KEGG pathway. GO terms and KEGG pathways with an adjusted *p* value less than 0.05 were considered significantly enriched by DEGs.

### Statistical analysis

For RNA-seq analysis, differential gene expression was determined using DESeq2 with an adjusted P-value <0.05 and log2 fold change >1 or < −1 as thresholds for significance. Data visualization and statistical evaluation were conducted using GraphPad Prism version 10.0. Results are presented as mean values ±standard error of the mean (SEM). Unpaired T-test was applied to assess differences across two groups. One-way analysis of variance (ANOVA) was applied to assess differences across multiple groups. Statistical significance is indicated as follows: ns (not significant), *p < 0.05, **p < 0.01.

## Results

### Structural characterization of the murine *Gatm* gene

**FIGURE 1:**
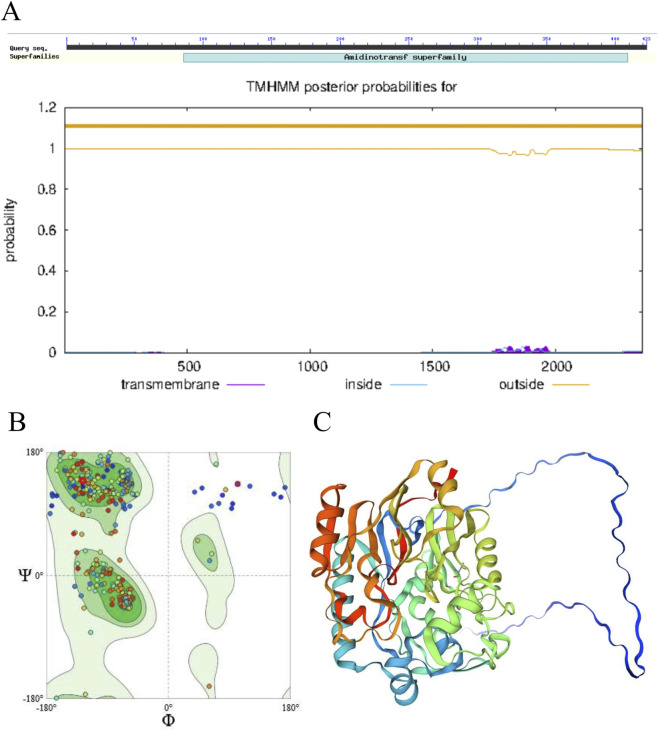
Structural characterization and conservation of the murine *Gatm* gene. **(A)** Schematic representation of the murine *Gatm* gene encoding a 423-amino acid protein. Conserved domain analysis identifies canonical amidinotransferase motifs and transmembrane topology prediction using TMHMM 2.0. **(B,C)** Three-dimensional structural modeling by SWISS-MODEL.

Bioinformatic analysis revealed that the murine *Gatm* gene (Gene ID: 67092) is located on chromosome 2 and consists of nine exons encoding a 423-amino acid protein (NP_080237.1). Conserved domain analysis identified canonical amidinotransferase motifs critical for cytosolic creatine synthesis, confirming functional homology between mouse and human GATM (Figure 1A). Transmembrane topology prediction using TMHMM 2.0 indicated no transmembrane helices, supporting the cytosolic localization of GATM consistent with its enzymatic role in the initial step of creatine biosynthesis. Structural modeling via SWISS-MODEL generated a high-confidence three-dimensional structure of mouse GATM, revealing strong conservation with the human ortholog, particularly within the catalytic core domain (Figures 1B,C). These analyses confirm the conserved genomic and structural architecture of *Gatm*, providing a solid foundation for *in vivo* functional studies.

### Generation and genotypic validation of *Gatm* knockout mice

**FIGURE 2:**
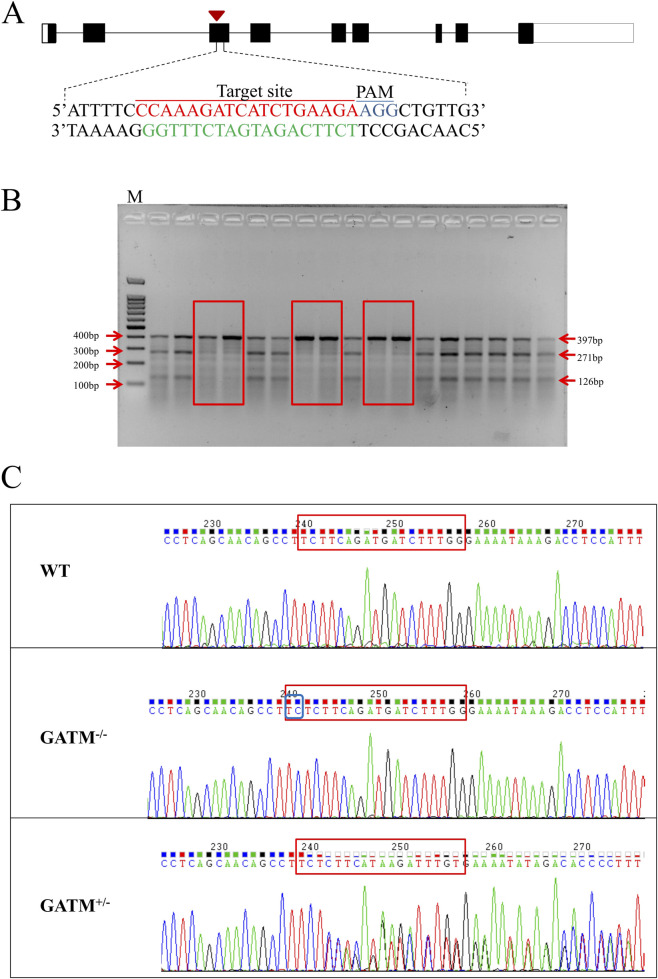
Generation and genotypic validation of *Gatm* knockout mice. **(A)** Targeting strategy using CRISPR/Cas9 to disrupt exon 3 of the *Gatm* gene. A single-guide RNA was designed to induce double-strand breaks at the indicated site. **(B)** Genotyping results showing PCR amplification followed by T7 Endonuclease I (T7EI) assay. Cleavage of the 397-bp amplicon into fragments of 271 bp and 126 bp confirms successful editing. **(C)** Sanger sequencing identifies a biallelic TC insertion in homozygous *Gatm* mice, resulting in a frameshift mutation and premature stop codon predicted to abolish functional GATM expression. Heterozygous (*Gatm*) and wild-type (WT) littermates were included as controls.

To investigate the physiological role of *Gatm* in spermatogenesis, we developed a constitutive knockout mouse model using CRISPR/Cas9. A single-guide RNA targeting exon 3 was designed (Figure 2A). Founder screening by PCR amplification and T7 Endonuclease I (T7EI) assay confirmed cleavage of the 397-bp amplicon into fragments of 271 bp and 126 bp, indicative heterozygosity (Figure 2B). Sanger sequencing identified a biallelic insertion (TC) at the target site in homozygous (*Gatm*) mice, resulting in a frameshift mutation and premature stop codon predicted to abolish functional GATM expression (Figure 2C). Heterozygous (*Gatm*) and wild-type (WT) littermates were genotyped in parallel to enable comparative analyses across genotypes. Throughout the study, we observed that *Gatm* ^−/−^ mice exhibited slightly reduced body size compared to age-matched WT and *Gatm* ^+/−^ controls; no other overt phenotypic abnormalities-beyond the reproductive defects-were observed.

### Testicular atrophy and histopathological abnormalities in *Gatm* mice

**FIGURE 3:**
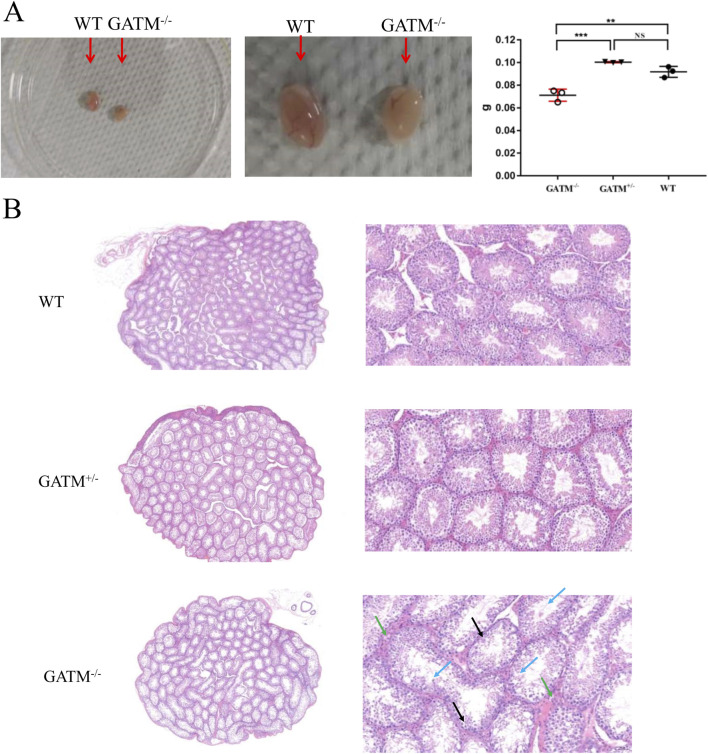
Testicular atrophy and histopathological defects in *Gatm*-deficient mice. **(A)** Testes from *Gatm* knockout males exhibit significantly reduced organ weight compared to heterozygous and WT controls. **(B)** Hematoxylin and eosin (H&E) staining of testicular sections. WT and *Gatm* mice show well-organized seminiferous tubules lined with four to five layers of germinal epithelium, abundant luminal spermatozoa, and normal Leydig cell morphology. In contrast, *Gatm* testes display severe thinning of the germinal epithelium, absence or scarcity of luminal sperm, arrested spermatogenesis at early stages, and interstitial edema, suggesting impaired spermatogenesis and potential vascular or immune dysregulation.

Phenotypic evaluation revealed severe reproductive defects in *Gatm* males. Testes from knockout animals exhibited significantly reduced organ weight compared to heterozygous and WT controls (Figure 3A), indicating marked testicular atrophy. Histological examination showed well-organized seminiferous tubules in WT and *Gatm* mice, lined with four to five layers of germinal epithelium, abundant luminal spermatozoa, and normal interstitial Leydig cell morphology (Figure 3B). In contrast, *Gatm* testes displayed pronounced thinning of the germinal epithelium—often limited to only two disorganized cell layers—with absent or sparse luminal sperm. Spermatogenesis was arrested at early stages, and interstitial edema was evident, suggesting potential vascular leakage or immune dysregulation (Figure 3B). These pathological changes collectively reflect a failure of spermatogenesis upon *Gatm* ablation.

### Ultrastructural sperm defects reveal cacospermia in *Gatm* mice

**FIGURE 4:**
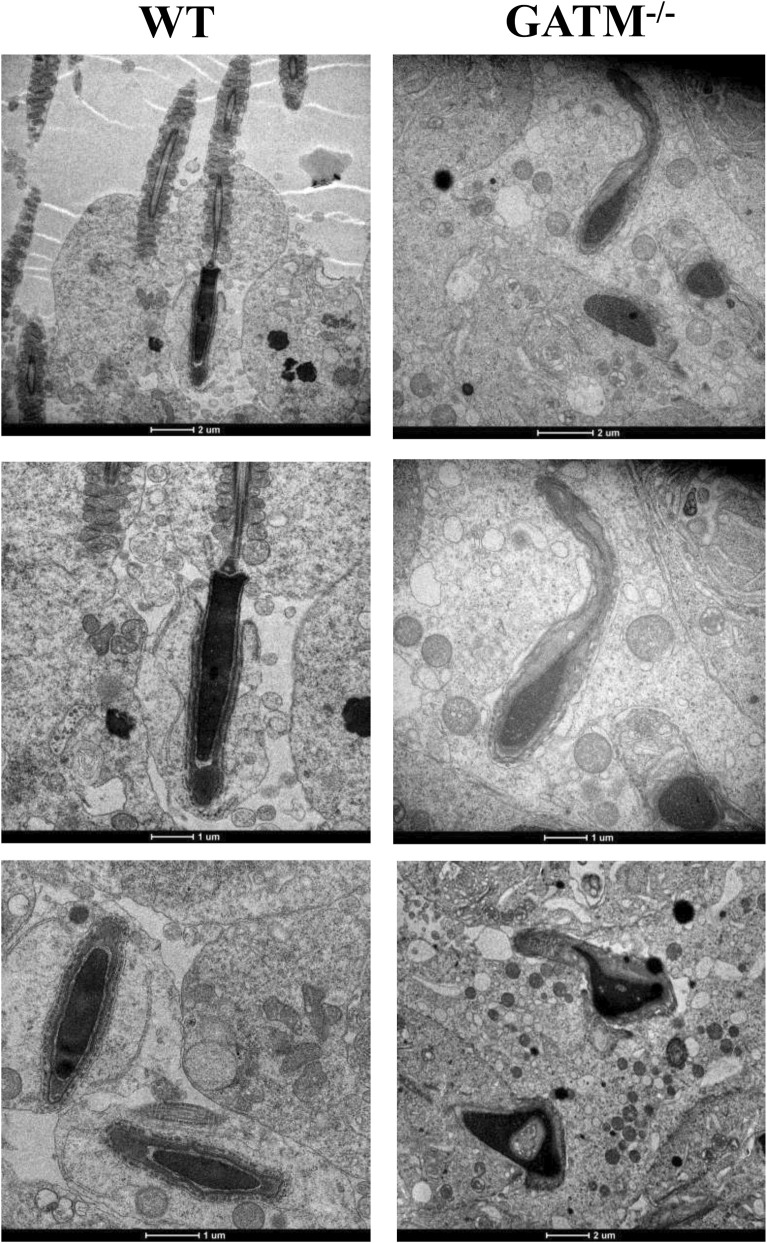
Ultrastructural sperm abnormalities in *Gatm* knockout mice reveal cacospermia. Transmission electron microscopy images of spermatozoa from wild-type (WT) and *Gatm*-deficient mice. In WT sperm, tightly packed mitochondrial sheaths uniformly surround the midpiece, and nuclei exhibit densely condensed chromatin. In contrast, *Gatm* sperm show marked depletion of mitochondria in the flagellar midpiece and pronounced nuclear vacuolization.

Transmission electron microscopy revealed severe ultrastructural defects in spermatozoa derived from *Gatm* knockout mice. In wild-type (WT) sperm, tightly packed mitochondrial sheaths uniformly surrounded the midpiece, and the nuclei displayed evenly condensed chromatin (Figure 4). In contrast, sperm from *Gatm*-deficient mice exhibited marked mitochondrial depletion in the flagellar midpiece and pronounced nuclear vacuolization (Figure 4). Furthermore, breeding experiments demonstrated that male mice with complete knockout of the *GATM* gene were infertile, whereas heterozygous *GATM* males showed normal fertility. These morphological abnormalities are consistent with the clinical definition of cacospermia—a condition characterized by abnormal sperm ultrastructure and impaired fertility. The disorganization of mitochondria likely disrupts localized ATP supply required for flagellar motility, while nuclear defects may compromise chromatin integrity and the sperm’s capacity to achieve successful fertilization. Collectively, these findings indicate that loss of *Gatm* leads to profound structural malformations in sperm, highlighting the critical role of creatine metabolism in maintaining proper sperm architecture.

### A hierarchical endocrine response to *Gatm* ablation in male mice

**FIGURE 5:**
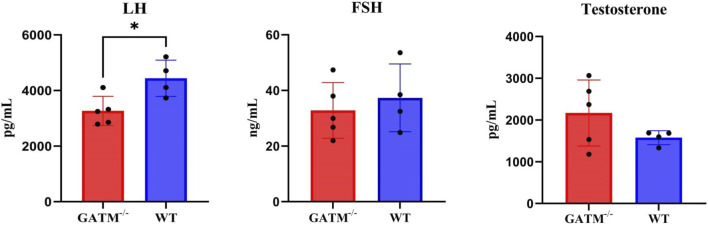
Serum hormone profiles in *Gatm* ^−/−^ and wild-type male mice. Statistical significance was determined by unpaired two-tailed Student’s t-test, *P* < 0.05 was considered statistically significant.

To determine whether endocrine disruption contributes to the spermatogenic arrest observed in *Gatm* ^−/−^ mice, we quantified serum levels of testosterone, follicle-stimulating hormone (FSH), and luteinizing hormone (LH) in archived samples from adult *Gatm* ^−/−^ and wild-type (WT) male mice. The results showed that serum LH levels were reduced in *Gatm* ^−/−^ mice by compared to WT controls (*p* = 0.01). In contrast, FSH showed a modest downward trend, but this difference did not reach statistical significance (*p* = 0.55). Serum testosterone displayed a upward trend in *Gatm* ^−/−^ mice by compared to the WT group, although this increase remained non-significant (*p* = 0.19) (Figure 5).

### Transcriptomic profiling reveals ribosomal and mitochondrial dysfunction in *Gatm* testicular tissues

**FIGURE 6:**
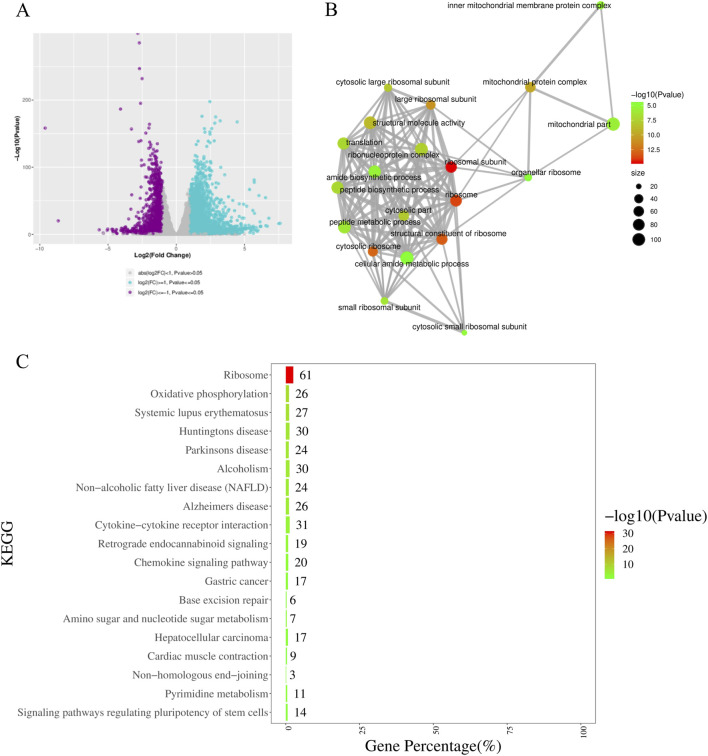
Transcriptomic profiling reveals ribosomal and mitochondrial dysfunction in *Gatm* testes. **(A)** Volcano plot showing 7,252 differentially expressed genes (DEGs; |log_2_FC| > 1, FDR <0.05) in whole testis tissue from *Gatm* vs. WT mice. **(B)** Gene Ontology (GO) enrichment analysis of downregulated genes highlights significant impairments in ribosome biogenesis, mitochondrial protein complex assembly, and ribosomal subunit organization. **(C)** KEGG pathway analysis confirms systemic disruption of ribosomal pathways.

To elucidate the molecular basis of testicular atrophy, we performed RNA-seq on whole testes from *Gatm* and WT littermates. Comparative analysis identified 7,252 differentially expressed genes (DEGs; |log_2_FC| > 1, FDR <0.05), including 4,701 upregulated and 2,551 downregulated transcripts (Figure 6A). Functional enrichment analysis of downregulated genes revealed significant impairment in key cellular processes: Gene Ontology (GO) terms such as *ribosome biogenesis*, *mitochondrial protein complex assembly*, and *ribosomal subunit organization* were highly enriched (Figure 6B). KEGG pathway analysis further confirmed systemic disruption of ribosomal pathways (Figure 6C), indicating widespread deficits in translational capacity.

**FIGURE 7:**
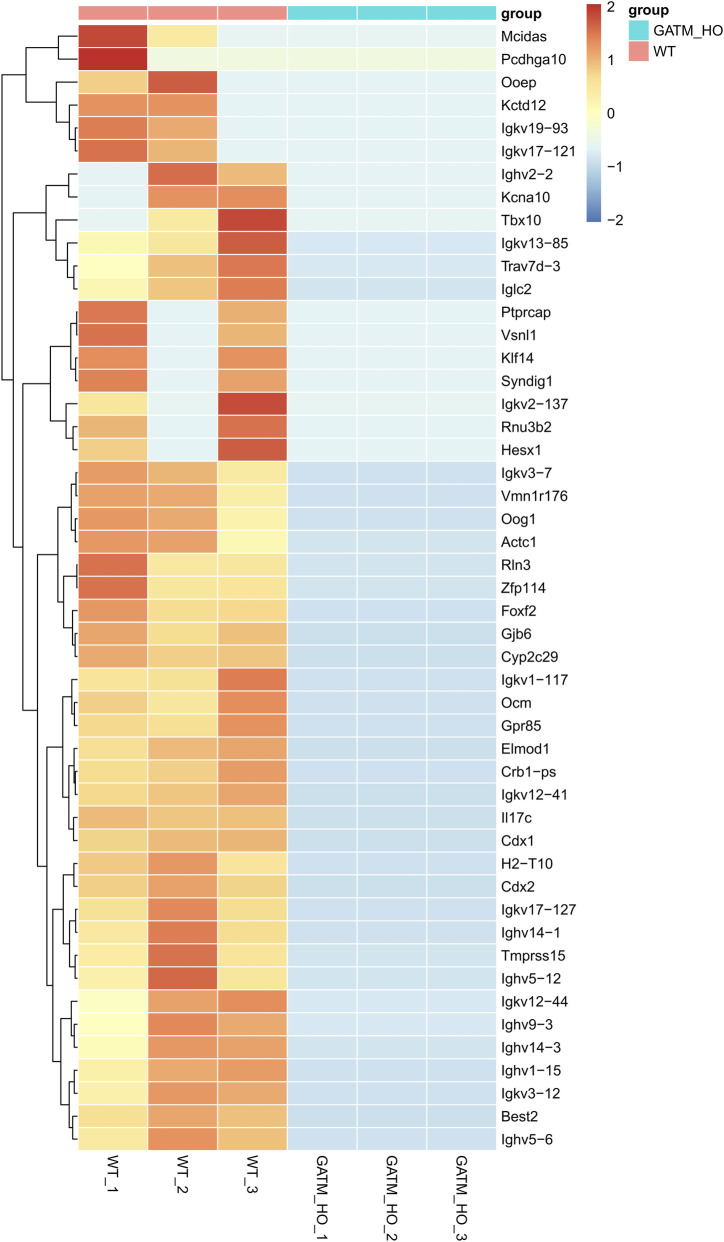
Transcriptional silencing of immunoglobulin variable region genes in *Gatm*-deficient testes. Heatmap showing expression levels of immunoglobulin heavy-chain variable (*Ighv*) and kappa-chain variable (*Igkv*) genes in WT and *Gatm* ^−/−^ testicular tissues.

Notably, we observed complete transcriptional silencing of multiple immunoglobulin variable region genes exclusively in *Gatm*-deficient testes. This included members of the immunoglobulin heavy-chain variable family (*Ighv*: e.g., *Ighv14-1*, *12–44*, *3–12*, *3–7*, *1–117*, *12–41*) and κ-chain variable genes (*Igkv*: e.g., *Igkv19-93*, *17–121*, *13–85*, *2–137*, *17–127*) (Figure 7). This observation suggests potential disruption of testicular immune microenvironment homeostasis following *Gatm* ablation.

### Sperm-specific transcriptome alterations indicate disrupted steroid biosynthesis and ion transport in *Gatm* mice

**FIGURE 8:**
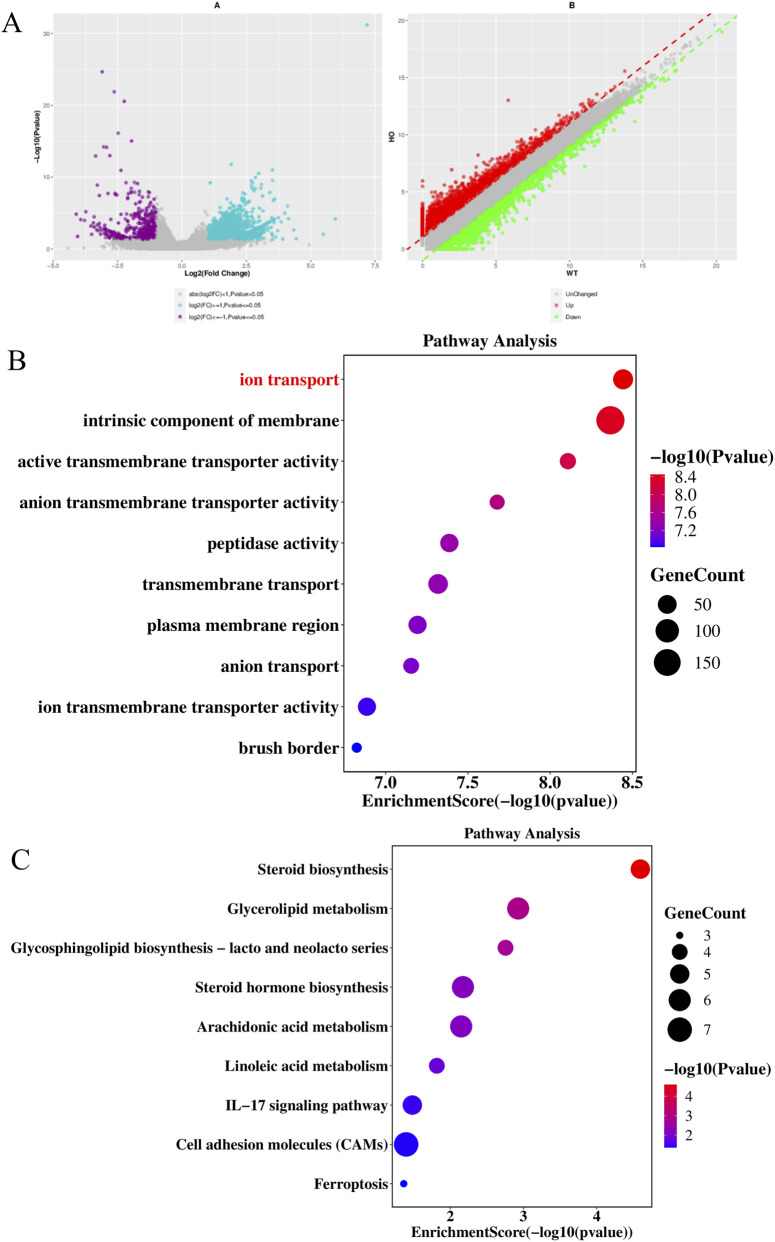
Sperm-specific transcriptome alterations in *Gatm* ^−/−^ mice reveal disrupted ion transport and steroid biosynthesis. **(A)** Volcano plot of differential gene expression in spermatozoa from *Gatm* ^−/−^ vs. WT mice (|log_2_FC| > 1, FDR < 0.05). **(B)** GO enrichment analysis of downregulated genes showing significant impairment in ion transport processes. **(C)** KEGG pathway analysis revealing downregulation of genes involved in steroid biosynthesis in *Gatm* ^−/−^ sperm.

We performed comparative transcriptome analysis on spermatozoa derived from Gatm^−/−^ (homozygous knockout), Gatm^+/−^ (heterozygous), and wild-type (WT) littermate mice. Differential gene expression (DGE) analysis identified 2,200 differentially expressed genes (DEGs) between Gatm^−/−^ and WT groups, comprising 1,683 upregulated and 517 downregulated transcripts (Figure 8A). Subsequent Gene Ontology (GO) term enrichment and KEGG pathway analysis of downregulated genes revealed significant biological implications. Specifically, GO analysis demonstrated marked downregulation of genes involved in ion transport etc., in Gatm^−/−^ sperms relative to WT controls (Figure 8B). Parallel KEGG analysis highlighted enrichment of downregulated genes in steroid biosynthesis pathways etc., suggesting disrupted lipid metabolism homeostasis (Figure 8C).

**FIGURE 9:**
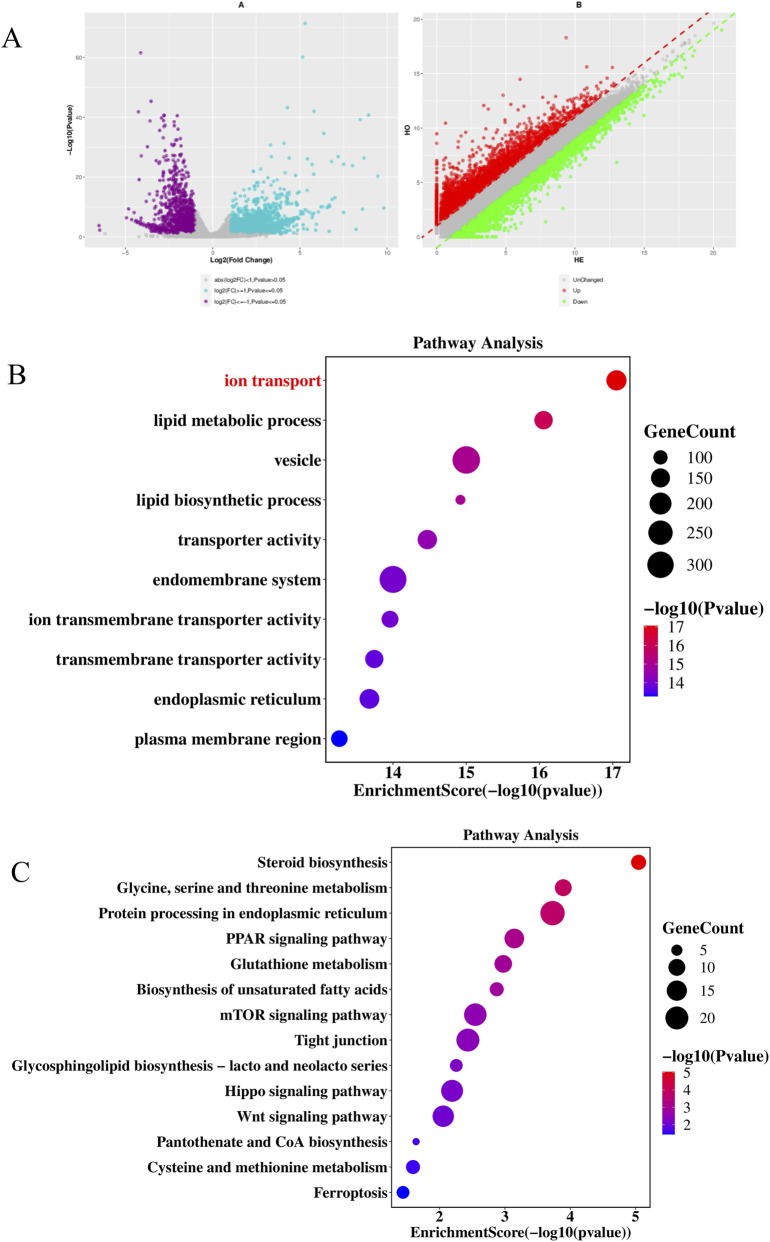
Comparative transcriptome analysis of *Gatm* ^−/−^ and *Gatm* ^+/−^ spermatozoa confirms dysregulation of steroidogenic and ion transport pathways. **(A)** Volcano plot of DEGs between *Gatm* ^−/−^ and *Gatm* ^+/−^ sperm (|log_2_FC| > 1, FDR < 0.05). **(B)** GO enrichment analysis of downregulated genes showing consistent impairment in ion transport. **(C)** KEGG pathway analysis again highlighting significant involvement of downregulated genes in steroid biosynthesis pathways.

In the comparative analysis between Gatm^−/−^ and Gatm^+/−^ cohorts, we identified 4,822 DEGs (3,247 upregulated; 1,575 downregulated) (Figure 9A). GO analysis of downregulated genes in this comparison corroborated the impairment of ion transport processes (Figure 9B), while KEGG pathway mapping again revealed predominant involvement in steroid biosynthesis (Figure 9C).

## Discussion

In this study, we identify GATM as a central orchestrator of spermatogenesis, integrating creatine-dependent energy metabolism with ribosomal biogenesis, mitochondrial integrity, and immune-related transcriptional regulation. Through a multidisciplinary approach combining genetic modeling, histopathology, ultrastructural imaging, and multi-compartment transcriptomics, we demonstrate that *Gatm* ablation leads to severe testicular atrophy, cacospermic sperm defects, and widespread molecular dysregulation—culminating in complete male infertility.

The conserved genomic and protein structure of murine GATM-featuring canonical amidinotransferase domains and cytosolic localization-confirms its essential role in the rate-limiting step of creatine synthesis (Ndika et al., 2012; Mulay and Anders, 2018; Baker et al., 2021). The exon 3-targeted frameshift knockout effectively abolishes functional GATM expression, enabling rigorous *in vivo* assessment of its reproductive functions. Phenotypically, *Gatm* mice exhibit testicular atrophy, disorganization of the seminiferous epithelium, and spermatogenic arrest-features closely resembling clinical manifestations of non-obstructive azoospermia in humans. The presence of interstitial edema further hints at possible disruption of vascular integrity or local immune homeostasis, warranting future investigation into stromal–germ cell crosstalk.

Ultrastructural analysis revealed classic hallmarks of cacospermia in *Gatm*-null sperm: mitochondrial depletion in the midpiece and nuclear vacuolization. These defects are incompatible with normal sperm motility or fertilization potential. Given the spatial separation between ATP production (mitochondria) and consumption sites (axoneme, plasma membrane), the phosphocreatine shuttle is thought to facilitate rapid, long-range energy distribution along the flagellum. Thus, GATM deficiency likely disrupts subcellular energy channeling, compromising both structural maturation and post-ejaculatory performance.

Transcriptomic profiling offers mechanistic insight into this bioenergetic collapse. In whole testis, *Gatm* loss elicits extensive transcriptional rewiring (7,252 DEGs), with notable downregulation of pathways governing ribosome biogenesis and mitochondrial complex assembly. Considering that late spermatids are transcriptionally silent, robust ribosomal production during earlier stages is crucial for sustaining the high translational demand of spermiogenesis. Its impairment likely contributes directly to failed differentiation and reduced sperm output. Moreover, the concurrent suppression of mitochondrial genes supports a dual-hit model: bioenergetic insufficiency arises not only from defective creatine-mediated ATP shuttling but also from diminished respiratory chain capacity.

Unexpectedly, RNA-seq revealed specific and complete silencing of multiple immunoglobulin variable region genes (*Ighv*, *Igkv*) in *Gatm*-deficient testes. Though classically linked to adaptive immunity, emerging evidence shows ectopic immunoglobulin expression in immune-privileged tissues—including the testis—where they may modulate local immune responses or act as stress sensors. Their selective repression in the absence of GATM implies a novel connection between cellular metabolism and transcriptional control of immune-modulatory elements. Whether this reflects altered Sertoli cell signaling, epigenetic reprogramming, or immune infiltration remains to be determined. Nevertheless, given that Sertoli cells maintain immune privilege through tight regulation of antigen presentation, disruption of immunoglobulin-like transcription might erode immune tolerance and exacerbate tissue injury—a hypothesis amenable to testing via spatial transcriptomics and immune cell profiling.

Sperm-specific transcriptomic analysis further uncovers autonomous consequences of *Gatm* loss. Downregulation of ion transport genes likely compromises membrane excitability and calcium signaling essential for capacitation and hyperactivation. The persistent suppression of steroid biosynthesis pathways—across both *Gatm* vs. WT and *Gatm* vs. *Gatm* comparisons—suggests systemic metabolic dysregulation. Although mature sperm lack *de novo* steroidogenic capability, residual cytoplasmic droplets may retain relevant transcripts, or these alterations may reflect developmental programming errors originating in earlier germ cell stages. Alternatively, chronic energy deficit could induce oxidative stress in Leydig cells, indirectly impairing androgen synthesis and thus sperm quality. The milder phenotype in heterozygotes supports a threshold effect, wherein partial GATM activity sustains baseline spermatogenesis under unstressed conditions but fails when metabolic demand increases. These findings resonate with broader themes in reproductive metabolism. Just as nicotinamide mononucleotide (NMN) supplementation improves oocyte quality during aging, our data raise the possibility that metabolic interventions—such as creatine supplementation, mitochondrial-targeted antioxidants might partially rescue the *Gatm*-deficient phenotype.

In summary, we redefine GATM as a pleiotropic regulator of male fertility whose influence extends far beyond its classical role in energy buffering. It coordinates translational capacity, organelle architecture, and potentially immune modulation—highlighting the exquisite metabolic sensitivity of spermatogenesis. Human *GATM* maps to chromosome 5q11.2 and encodes a highly conserved enzyme, raising the prospect that hypomorphic variants or regulatory polymorphisms may contribute to idiopathic male infertility. Future work should explore tissue-specific rescue models and evaluate GATM expression and creatine metabolite levels in patient cohorts with unexplained oligozoospermia or azoospermia. Such efforts may pave the way for precision diagnostics and targeted therapies for metabolic subtypes of male reproductive failure.

## Data Availability

The original sequencing data that support the findings of this study have been deposited into CNSA (https://db.cngb.org/) with accession number CNP0009127.

## References

[B1] Baker S. A. Gajera C. R. Wawro A. M. Corces M. R. Montine T. J. (2021). GATM and GAMT synthesize creatine locally throughout the mammalian body and within oligodendrocytes of the brain. Brain Res. 1770, 147627. 10.1016/j.brainres.2021.147627 34418357

[B2] Carney E. F. (2018). GATM mutations cause mitochondrial abnormalities and kidney failure. Nat. Rev. Nephrol. 14, 414. 10.1038/s41581-018-0017-3 29695752

[B3] Forst A.-L. Reichold M. Kleta R. Warth R. (2021). Distinct mitochondrial pathologies caused by mutations of the proximal tubular enzymes EHHADH and GATM. Front. Physiol. 12, 715485. 10.3389/fphys.2021.715485 34349672 PMC8326905

[B4] Kang X. Yan L. Wang J. (2024). Spatiotemporal distribution and function of mitochondria in oocytes. Reprod. Sci. 31, 332–340. 10.1007/s43032-023-01331-8 37605038

[B5] Li R. Albertini D. F. (2013). The road to maturation: somatic cell interaction and self-organization of the mammalian oocyte. Nat. Rev. Mol. Cell Biol. 14, 141–152. 10.1038/nrm3531 23429793

[B6] Liao X. Zhou S. Zeng D. Ying W. Lian D. Zhang M. (2023). Roles of the crucial mitochondrial DNA in hypertrophic cardiomyopathy prognosis and diagnosis: a review. Med. Baltim. 102, 102. 10.1097/MD.0000000000036368 38050313 PMC10695538

[B7] Liu C.-C. (2025). Mitochondrial gene regulation and pain susceptibility: a multi-omics causal inference study. Int. J. Mol. Sci. 26, 8690. 10.3390/ijms26178690 40943610 PMC12428822

[B8] Liu B. Gao X. Teng H. Zhou H. Gao B. Li F. (2024). Association between GATM gene polymorphism and progression of chronic kidney disease: a mitochondrial related genome-wide Mendelian randomization study. Sci. Rep. 14, 20346. 10.1038/s41598-024-68448-x 39284843 PMC11405879

[B9] Liu D. Guo K. Li M. Yu X. Guan X. Guan X. (2025). The role of mitochondria-related genes and immune infiltration in carotid atherosclerosis: identification of hub targets through bioinformatics and machine learning approaches. Front. Genet. 16, 1597445. 10.3389/fgene.2025.1597445 40836955 PMC12361237

[B10] Liu S. Huang L. Lin L. Shan H. Wan Y. (2026). Identification and validation of critical mitochondrial hub genes for prostate cancer. Oncol. Lett. 31, 66. 10.3892/ol.2025.15419 41415479 PMC12709149

[B11] Medeiros M. A. Abreu B. J. Lima JPMS (2025). Assessing creatine-related gene expression in kidney disease: can available data give insights into an old discussion? Nutrients 17, 651. 10.3390/nu17040651 40004980 PMC11858045

[B12] Mulay S. R. Anders H.-J. (2018). Genetic mitochondrial glycine amidinotransferase protein aggregate formation triggers microparticle sensing and kidney failure. Ann. Transl. Med. 6, 315. 10.21037/atm.2018.07.09 30364060 PMC6186980

[B13] Ndika J. D. T. Johnston K. Barkovich J. A. Wirt M. D. O’Neill P. Betsalel O. T. (2012). Developmental progress and creatine restoration upon long-term creatine supplementation of a patient with arginine:glycine amidinotransferase deficiency. Mol. Genet. Metabolism 106, 48–54. 10.1016/j.ymgme.2012.01.017 22386973

[B14] Peng J. Ramatchandirin B. Pearah A. Maheshwari A. He L. (2022). Development and functions of Mitochondria in early life. Newborn Clarksv. 1, 131–141. 10.5005/jp-journals-11002-0013 37206110 PMC10193534

[B15] Placidi M. Emidio G. D. Virmani A. D’Alfonso A. Artini P. G. D’Alessandro A. M. (2022). Carnitines as mitochondrial modulators of oocyte and embryo bioenergetics. Antioxidants, 11 (4), 745. 10.3390/antiox11040745 35453430 PMC9024607

[B16] Qin W. Kutny P. M. Maser R. S. Dion S. L. Lamont J. D. Zhang Y. (2016). Generating mouse models using CRISPR‐Cas9‐Mediated genome editing. CP Mouse Biol. 6, 39–66. 10.1002/9780470942390.mo150178 26928663 PMC4848752

[B17] Schirrmacher V. (2020). Mitochondria at work: new insights into regulation and dysregulation of cellular energy supply and metabolism. Biomedicines 8, 8. 10.3390/biomedicines8110526 33266387 PMC7700424

[B18] Shriver L. P. Polacco B. J. Eckhardt M. Chang M. W. Martínez-Romero C. Adkins-Travis K. (2025). Integrative multi-omics analysis *in vivo* identifies influenza virus host factors. iScience 28, 113644. 10.1016/j.isci.2025.113644 41142120 PMC12552919

[B19] Wallimann T. Tokarska-Schlattner M. Schlattner U. (2011). The creatine kinase system and pleiotropic effects of creatine. Amino Acids 40, 1271–1296. 10.1007/s00726-011-0877-3 21448658 PMC3080659

[B20] Wang H. Yang H. Shivalila C. S. Dawlaty M. M. Cheng A. W. Zhang F. (2013). One-step generation of mice carrying mutations in multiple genes by CRISPR/Cas-mediated genome engineering. Cell 153, 910–918. 10.1016/j.cell.2013.04.025 23643243 PMC3969854

[B21] Wei B.-H. Hao S.-L. Yang W.-X. (2022). Regulation of spermatogonial stem cell self-renewal and proliferation in mammals. Histol. Histopathol. 37, 825–838. 10.14670/HH-18-461 35470414

[B22] Yu L. Wang L. Hu G. Ren L. Qiu C. Li S. (2022). Reprogramming alternative macrophage polarization by GATM-mediated endogenous creatine synthesis: a potential target for HDM-induced asthma treatment. Front. Immunol. 13, 937331. 10.3389/fimmu.2022.937331 36177049 PMC9513582

[B23] Zhu Z. Wang W. Zhang Q. Bi X. Ma S. Shen Y. (2025). Integration of multiple omics reveals key targets and cellular mechanisms for intervention in sarcopenia. Arch. Gerontol. Geriatr. 142, 106113. 10.1016/j.archger.2025.106113 41389732

