# Supplementary material for: Gatm ablation disrupts spermatogenesis by impairing ribosome biogenesis and coupling defective steroid biosynthesis to immunoglobulin silencing
Source: Front Cell Dev Biol. 2026 Mar 12;14:1786659. doi: 10.3389/fcell.2026.1786659 (PMC13017889; doi:10.3389/fcell.2026.1786659)
Supplement: Supplementary file 1 [file DataSheet1.docx]

**Supplementary materials of** **generation and genotyping of *Gatm* gene knockout mice**

Gene targeted sites for mouse Gtam were analyzed by the CHOPCHOP (http://chopchop.cbu.uib.no/). The gene targeted sites without predicted off-targeted sites and with highest score were chosen. The methods of generation gene knockout mice by using CRISPR/Cas9 system were described as previous publication. Briefly, 4-6 week old female C57BL/6J mice were superovulated by intraperitoneal injection of 5 IU pregnant mare serum gonadotropin (PMSG; Sigma-Aldrich, St. Louis, MO, USA), followed 48 hours later by 5 IU human chorionic gonadotropin (hCG; Sigma-Aldrich). Immediately after hCG injection, females were housed with males at a 1:1 ratio. Fertilized zygotes were harvested from the oviducts 16-18 hours post-hCG injection and collected from the oviducts of female mice. The entire oviduct with a small segment of the uterus was excised and placed in M2 medium. Under a dissecting microscope, the swollen ampulla of the oviduct was located, and the zygotes surrounded by cumulus cells were released by tearing the ampulla with fine-tipped forceps. Sometimes, fertilized zygotes may migrate from the ampulla to the narrow part of the oviduct. In such cases, a flushing method was used. A 1 ml syringe with a blunted 4-gauge needle was prepared, preheated to 37℃ medium was drawn into the syringe. The excised oviduct was placed in a 6 cm petri dish, and the needle was inserted into the infundibulum. The infundibulum was gently clamped with forceps to fix the needle and prevent slipping. The syringe was slowly pushed to allow the zygotes to flow out from the uterine end along with the medium. Subsequently, the fertilized zygotes with cumulus cells were transferred to M2 medium containing 1 mg/ml Hyaluronidase to remove the cumulus cells. Immediately, the zygotes were aspirated into fresh M2 medium using a pre-pulled pipette and washed 2-3 times. Then, the zygotes were transferred to another fresh M2 medium and washed 4-5 times in the same way until no tissue debris or blood cells were visible in the field of view. Finally, the fertilized zygotes were transferred to CO2-equilibrated M16 medium drops (approximately 50 μl), which were overlaid with a layer of embryo-grade paraffin oil, and cultured in an incubator. Microinjection was performed when the pronuclei developed clearly and plumply. After microinjection, the fertilized zygotes were placed back into M16 medium and continued to be cultured in the cell incubator. The next batch of fertilized zygotes was aspirated for injection until all injections were completed. The injected fertilized zygotes were cultured overnight for subsequent transfer into the oviducts of surrogate mice. Surrogate mice were usually selected from estrous CD1 female mice (over 6 weeks old, preferably parous) that were caged with vasectomized male mice. The next morning, mating status was checked, and those with vaginal plugs were used as surrogate mice. First, two-cell stage embryos were transferred from M16 medium to M2 medium using an embryo transfer pipette and washed once or twice. A new transfer pipette was used to aspirate a small segment of paraffin oil and a little M2 medium, followed by 10-15 microinjected two-cell embryos. Surrogate mice were anesthetized by intraperitoneal injection of 10% chloral hydrate at a dose of 5 ml/kg. After deep anesthesia, the dorsal fur was cut with iris scissors and disinfected by wiping with 75% alcohol. A longitudinal incision of about 1 cm was made 1 cm beside the intersection of the posterior midline and the horizontal line at the costal margin, and a white fat pad was visible. The fat pad was pulled out with curved iris forceps to expose the ovary, under which was the oviduct. Under a microscope, the membrane between the oviduct and ovary was carefully torn with micro forceps to find the opening of the oviduct. The embryo transfer pipette was quickly inserted into the opening, and the embryos were blown in, while avoiding blowing in too much medium and paraffin oil. The same method was used for embryo transfer into the other oviduct. After surgery, the pseudopregnant mice were kept warm until they woke up, and then properly reared.

The mixture of Cas9 mRNA (100 ng/ m L) and gRNA (50 ng/ m L) were co-injected into the cytoplasm of the zygotes with visible pronuclei by using the Leica DMI8 inverted microscope with the eppendorf microinjection system (FemtoJet® 4i/ FemtoJet® 4x). The F0 founders and their next generations were genotyping by PCR, T7E1 analysis and Sanger sequencing. More specifically, genomic DNA from mouse tail tissues were extracted by EasyPure Genomic DNA Kit (TransGen, No.EE101). Total 100 ng genomic DNA was used for PCR amplification with primers flanking the targeted site. 10 μl of each PCR product was heat-denatured, re-annealed, and digested with T7 Endonuclease I (New England Biolabs). The samples were analyzed by 2% agarose/1×TAE gel electrophoresis. The PCR products were purified by using Wizard ® SV Gel and PCR Clean-Up System (Promega, No. A9281) and were directly sent for Sanger sequencing by specific primers in company (BioSune, Shanghai, China). All [primer](javascript:;)s were [synthesized](javascript:;) in company (BioSune, Shanghai, China). The total time required to generate and genotype the stable Gatm^-/-^ line was approximately 8-12 months, including embryo microinjection, F0 generation breeding, F1 genotyping, and establishment of homozygous breeding colonies. For breeding, we adopted an optimized strategy to generate experimental animals: GATM⁻/⁻ females were crossed with GATM⁺/⁻ males. This cross yields, on average, 50% GATM⁻/⁻ and 50% GATM⁺/⁻ offspring. GATM⁻/⁻ male mice were then selected from the GATM⁻/⁻ offspring cohort for downstream experiments.
